# Plasmonic meniscus lenses

**DOI:** 10.1038/s41598-022-04954-0

**Published:** 2022-01-18

**Authors:** Joseph Arnold Riley, Noel Healy, Victor Pacheco-Peña

**Affiliations:** 1 School of Mathematics, Statistics and Physics, Newcastle University, Newcastle Upon Tyne, NE1 7RU UK; 2 School of Engineering, Newcastle University, Newcastle Upon Tyne, NE1 7RU UK

**Keywords:** Nanophotonics and plasmonics, Integrated optics

## Abstract

Controlling and manipulating the propagation of surface plasmons has become a field of intense research given their potential in a wide range of applications, such as plasmonic circuits, optical trapping, sensors, and lensing. In this communication, we exploit classical optics techniques to design and evaluate the performance of plasmonic lenses with meniscus-like geometries. To do this, we use an adapted *lens maker equation* that incorporates the effective medium concepts of surface plasmons polaritons travelling in dielectric-metal and dielectric-dielectric-metal configurations. The design process for such plasmonic meniscus lenses is detailed and two different plasmonic focusing structures are evaluated: a plasmonic lens with a quasi-planar output surface and a plasmonic meniscus lens having a convex-concave input–output surface, respectively. The structures are designed to have an effective focal length of 2*λ*_0_ at the visible wavelength of 633 nm. A performance comparison of the two plasmonic lenses is shown, demonstrating improvements to the power enhancement, with a 22% and 16.5% increase when using 2D (ideal) or 3D (realistic plasmonic) meniscus designs, respectively, compared to the power enhancement obtained with convex-planar lenses. It is also shown that the depth of focus of the focal spot presents a 19.8% decrease when using meniscus lenses in 2D and a 34.3% decrease when using the proposed 3D plasmonic meniscus designs. The broadband response of a plasmonic meniscus lens (550–750 nm wavelength range) is also studied along with the influence of potential fabrication errors on the generated effective focal length. The proposed plasmonic lenses could be exploited as alternative focusing devices for surface plasmons polaritons in applications such as sensing.

## Introduction

Plasmonics research and innovation has grown at an unprecedented speed within the past decade enabling the manipulation of light-matter interactions at nanometer scales^1^. Plasmonic structures have the advantages of both the compactness of electronics and the data-carrying capacity of photonics^2^, opening them to a broad range of applications such as focusing^3–6^, sensing^7,8^, plasmonic circuitry^9–12^ and optical trapping^13^ among others. Surface Plasmon Polaritons (SPPs) are surface electromagnetic waves that propagate along a dielectric-metal interface. They are excited when photons couple with the collective conduction electrons in a metal at optical frequencies resulting in bound oscillations. These surface waves are strongly confined at the dielectric-metal interface^1,14^ and decay as they propagate due to intrinsic metallic losses at optical frequencies. In this context, controlling and manipulating their propagation is crucial if one aims to exploit them for use in ultra-compact devices and systems^15^.

The field of lenses has also benefited greatly from the research of plasmonics and the manipulation of SPPs where different techniques have been proposed to design focusing devices, such as metalenses for free-space focusing^16^, the excitation of photonic nanojets from cuboid and cylinder structures^5,17,18^, as well as broadband achromatic lenses for SPP focusing^3,19^, among others. In this context, the design of SPP-based focusing devices can be carried out by borrowing and applying classical and transformation optics techniques to plasmonic structures^4,20,21^. This has led to the demonstration of analogous plasmonic devices for the manipulation and focusing of SPPs such as double convex designs^3^, plasmonic Fresnel lenses^22^ as well as Eaton and Luneburg lenses^23^.

As it is known, lenses can be designed using the *lens maker equation* where a focus is determined using the radii of curvature of each side (input and output surfaces) of the lens, the lens thickness, and the refractive index of both the lens and the surrounding medium^24^. By varying these parameters, different lens profiles can be obtained such as double-convex/concave, meniscus, and plano-convex/concave lenses^24,25^. As can be expected, each lens profile possesses particular properties such as a positive or negative focal length (where the lens behaves as a converging or diverging device, respectively) as well as different spatial resolution capabilities^26^. Though developed in classical optics, this technique has been exploited in different scenarios for SPP focusing such as in gradient index^27^ and biconvex^3^ plasmonic lenses. In this work, we ask if it is possible to design plasmonic lenses with a meniscus shape using techniques taken from classical optics? In general meniscus lenses at millimeter/microwave frequencies (when considering free-space designs illuminated with a plane wave), offer enhanced spatial resolution of the focus and reduced chromatic aberrations^28,29^. Here, we explore if this improvement extends to plasmonic analogues by implementing meniscus profiles as alternative geometries to more commonly used designs such as convex-planar or biconvex focusing structures.

**Figure 1:**
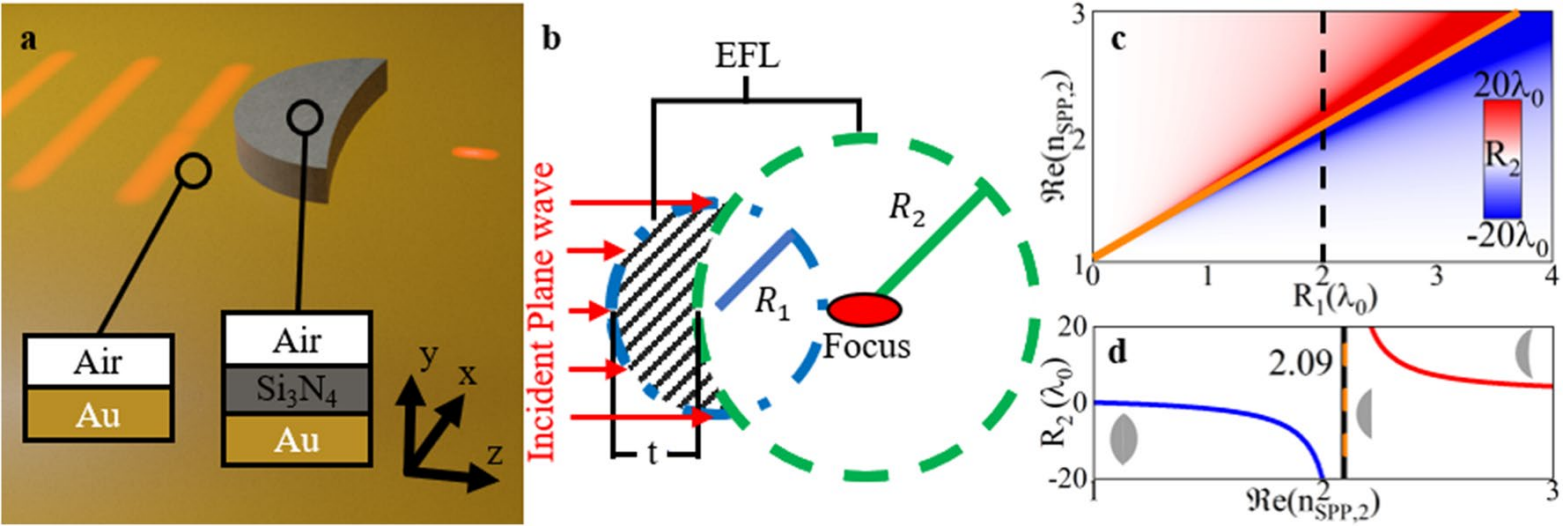
(**a**) Artistic representation of the proposed plasmonic meniscus lens. The insets represent the material compositions of the two regions where region 1 is air-Au (IM) and region 2 is air-Si_3_N_4_-Au (IIM). (**b**) Schematic representation of the *lens maker equation* used to design the plasmonic meniscus lenses, where *R*_1_ and *R*_2_ are the radii of curvature of the input and output surfaces of the lens, respectively, *t* is the lens thickness along the *z*-axis at *x* = *y* = 0 and *EFL* is the effective focal length, the length from the second principal plane of the lens to the maximum of the focus. (**c**) Analytical values of *R*_2_ representing the transition from negative values (blue), biconvex lens, to infinity (orange line), convex-planar, to positive values (red), meniscus, as *R*_1_ and $\Re e\left( {n_{SPP,2} } \right)$ vary. The colour bar inset represents the values of *R*_2_. The black dashed line shows when *R*_1_ = 2*λ*_0_. (**d**) Value of *R*_2_ along the dashed black line in (**c**) considering *R*_1_ = 2*λ*_0_.

In this work, we aim to answer these questions by proposing and studying two different plasmonic lenses designed using the *lens maker equation* along with effective medium concepts for SPPs^1^. For the first lens, the focusing structure is designed to have a convex-planar shape by selecting a relatively small effective refractive index (*n*_*SPP*_) for the SPPs traveling inside of the dielectric–dielectric–metal region. For the second plasmonic lens, we increased the value of *n*_*SPP*_ to generate a positive meniscus lens. The plasmonic lenses are designed at the wavelength of *λ*_0_ = 633 nm with a focus at an effective focal length (*EFL*, defined as the length from the second principal plane of the lens to the maximum of the focus, as shown in Fig. 1b) of *EFL* = 2*λ*_0_ and their performance is numerically evaluated using the commercial software COMSOL Multiphysics. The SPP focusing structures are studied in terms of the power enhancement at the focal spot, depth of focus, and spatial resolution along the transverse axis. Moreover, to evaluate the *lens maker equation* technique for the design of plasmonic meniscus lenses, we study the broadband response of a meniscus lens. The focusing device is evaluated in terms of its *EFL* and power enhancement at the focal spot, demonstrating a good agreement with the analytically calculated values found directly from the *lens maker equation*. These studies will demonstrate the potential that plasmonic meniscus lenses may have in applications such as sensors.

## Design

### Dispersion relations of SPPs

To begin, a schematic representation of the plasmonic lens under study for controlling SPPs and to achieve focusing is shown in Fig. 1a along with the design parameters represented in Fig. 1b. The plasmonic structure consists of a shaped dielectric block of silicon nitride (Si_3_N_4_), which will act as the plasmonic lens, placed on top of a semi-infinite metallic slab of gold (Au) with a dispersive permittivity modelled using Johnson and Christy’s experimental data^30^. We consider that the whole structure is immersed in air as the surrounding material (*n*_0_ = 1). The plasmonic lenses are designed at the telecommunications wavelength of *λ*_0_ = 633 nm. At this wavelength, the refractive index of Si_3_N_4_ is ~ 2.01^31^. Note that different dielectrics such as poly(methyl methacrylate) (PMMA)^23^ could also be implemented. Here, we use Si_3_N_4_ as it is a widely used material in plasmonics with a relatively high refractive index, allowing the design of compact plasmonic devices. The SPPs are excited from the back of the plasmonic lenses using narrow slits carved within the Au block^32,33^. With this setup, the SPPs can propagate along the positive *z*-direction at the air-Au interface with the electric field having *y*- and *z*-components while the magnetic field is parallel to the *x*-axis (i.e., a TM wave)^1,5^.

To be able to manipulate the SPPs in this configuration, first it is important to describe the properties of the different regions where the SPPs will propagate. The process to determine the properties of the different effective media along the path where the SPPs will travel has been used in the past for the design of plasmonic devices such as beam steerers^34^ and Eaton lenses^23^ but we will briefly describe the fundamental features for completeness. As can be seen in the insets of Fig. 1a, there are two different regions depending on the materials involved: here we name region 1 the Insulator–Metal (IM) interface (air–Au in our case), while region 2 has a sandwiched dielectric, Si_3_N_4_, between the two semi-infinite air and Au media, i.e., an Insulator–Insulator–Metal (IIM) region, or air–Si_3_N_4_–Au in our case.

With this configuration, the Si_3_N_4_ layer from the latter region is shaped to act as the plasmonic lens. For region 1, the complex effective refractive index of the SPPs can be calculated using the well-known expression for SPPs traveling at the interface between two semi-infinite media (a dielectric and a metal) $n_{SPP,1} = \sqrt {\frac{{n_{1}^{2} n_{Au}^{2} }}{{n_{1}^{2} + n_{Au}^{2} }}}$, where *n*_1_ is the refractive index of the semi-infinite dielectric in region 1, which in our designs is air (*n*_1_ = *n*_0_ = 1), and *n*_*Au*_ is the refractive index of the bottom semi-infinite metal (Au in our case as explained above)^5,34^. To calculate the complex effective refractive index in region 2 (IIM region with air–Si_3_N_4_–Au) we first calculate the propagation constant (*β*_*SPP*,2_) for the SPPs propagating in an IIM configuration. This can be analytically calculated by numerically solving the following transcendental equation:^23^

1 $$\begin{array}{*{20}c} {\tanh \left( {k_{2} l_{y} } \right) = - \left( {\frac{{\epsilon_{Au} \epsilon_{2} k_{2} k_{1} + \epsilon_{1} \epsilon_{2} k_{Au} k_{2} }}{{\epsilon_{Au} \epsilon_{1} k_{2}^{2} + \epsilon_{2}^{2} k_{Au} k_{1} }}} \right)} \\ \end{array}$$

where $k_{i} = \left( {\beta_{SPP,2}^{2} - \epsilon_{i} k_{0}^{2} } \right)^{\frac{1}{2}}$ is the wavenumber, $\epsilon_{i}$ is the complex permittivity with the subscript *i* = 1, 2 and Au representing each medium: air, Si_3_N_4_ and Au, respectively, and *l*_*y*_ is the height of the Si_3_N_4_ dielectric in the *y*-direction. Once the effective propagation constant for the SPPs in region 2 (*β*_*SPP*,2_) is obtained, the complex effective refractive index in the region 2 (*n*_*SPP*,2_) can be calculated by simply defining the ratio of *β*_*SPP*,2_ and the wave number in free space $\left( {k_{0} = \frac{{\lambda_{0} }}{2\pi }} \right)\,{\text{as}}\,n_{SPP,2} = \frac{{\beta_{SPP,2} }}{{k_{0} }}$^1,34^. From this expression along with Eq. (1), it can be observed how *n*_*SPP*,2_ is explicitly dependent on the height of the dielectric used (*l*_*y*_), as expected^5,6,34^. Hence one can tune this parameter to design IIM regions having tailored effective values of *n*_*SPP*,2_.

### Plasmonic lens designs: the lens maker equation

Now that the propagation properties of SPPs for region 1 have been defined and the means to determine the propagation features in region 2 have been obtained, we can now introduce the *lens maker equation* as a general formulation for the design of lenses. Such a formulation has been applied in multiple scenarios such as bi-convex/concave, plano-convex/concave, and positive/negative meniscus lenses working at microwave frequencies, demonstrating to be a powerful tool for the design of optical focusing elements^24^. In this context, as we aim to study such techniques for plasmonic meniscus lenses, the adapted full *lens maker equation* can be mathematically defined as follows (see a schematic representation of the parameters of the lens in Fig. 1b):^24^

2 $$\begin{array}{*{20}c} {\frac{1}{EFL} = \left( {\Phi - 1} \right)\left[ {\frac{1}{{R_{1} }} - \frac{1}{{R_{2} }} + \frac{{\left( {\Phi - 1} \right)t}}{{\Re e\left( {n_{SPP,2} } \right)R_{1} R_{2} }}} \right]} \\ \end{array}$$

where *EFL* is the effective focal length, $\Phi = \frac{{\Re e\left( {n_{SPP,2} } \right)}}{{\Re e\left( {n_{SPP,1} } \right)}}$ is the ratio between the real part of the effective refractive index of SPPs traveling in region 2 (IIM) and region 1 (IM), *R*_1_and *R*_2_ are the radii of curvature of the input and output surfaces of the lens, the left and right faces of the lens in Fig. 1a, respectively, and *t* is the thickness of the lens along the *z*-direction at *x* = *y* = 0. Note that, differently than the free-space classical lens-maker equation^24^ that requires Φ = *n*_2_/*n*_1_ with *n*_1,2_ as the refractive index of air and the lens respectively, in our case the refractive index of the region outside (air-Au) the plasmonic lens is *n*_*SPP*,1_ ≠ 1. Hence, we consider the full ratio $\Phi = \frac{{\Re e\left( {n_{SPP,2} } \right)}}{{\Re e\left( {n_{SPP,1} } \right)}}$ in Eq. (2). For cases where *t* < <*|R*_*1*_*|, |R*_*2*_*|*, *|R*_*1*_*-R*_*2*_*|* a simplified version of the *lens maker equation* can be used however for the following work we will proceed with the *full lens maker equation* for completeness as for meniscus profiles this requirement is not met as we are working with *R*_*1*_ and *R*_*2*_ values of the same order as the lens thickness.

As shown in Eq. (2), the lens profile depends on multiple parameters. For instance, let us consider a fixed value for the *EFL* being 2*λ*_0_. In this scenario, we need to define the parameters *R*_1_, *R*_2_, *t* and *n*_*SPP*_. As the *n*_*SPP*_ in each region (1 and 2) is known (as described in the previous section) we only need to define *t*, *R*_1_ and *R*_2_. To produce a positive meniscus lens (i.e., a converging lens) we require both *R*_1_ and *R*_2_ to be positive. This condition can be explained by rearranging Eq. (2) to calculate *R*_2_, as follows:

3 $$\begin{array}{*{20}c} {R_{2} = \frac{{ - \Re e\left( {n_{SPP,2} } \right) \Phi\, EFL\, R_{1} + \Re e(n_{SPP,2} ) \,EFL\, R_{1} + \Phi^{2} t \,EFL\, - 2\Phi t\, EFL + t \,EFL}}{{\Re e\left( {n_{SPP,2} } \right)\left( {EFL\, + R_{1} - \Phi\, EFL} \right)}}} \\ \end{array}$$

As observed from the expression above, one can simply modify *R*_1_ and $\Re e\left( {n_{SPP,2} } \right)$ using practical values (i.e., values of $\Re e\left( {n_{SPP,2} } \right)$ calculated using Eq. (1) considering available materials for the dielectric and metallic regions as described above) and calculate the unknown *R*_*2*_. We provide an example in Fig. 1c where we consider *λ*_0_ = 633 nm, *EFL* = 2*λ*_0_, *t* = 600 nm (~ 0.95λ_0_) and $\Re e\left( {n_{SPP,1} } \right) = 1.045$, calculated as described in the previous section (we will use the same parameter of *EFL* and $\Re e\left( {n_{SPP,1} } \right)$ for the designs of the plasmonic lenses in the following sections). For completeness, we show in Fig. 1d, a case where *R*_1_ is fixed to be *R*_1_ = 2*λ*_0_ (values extracted from the vertical dashed line from Fig. 1c). As it can be observed in Fig. 1c,d, three different geometrical shapes of the output surface of the lens can be obtained when considering values of *R*_1_ > 0: i) when *R*_2_ < 0 (shown as the blue region in Fig. 1c and values of $\Re e\left( {n_{SPP,2} } \right) < 2.09$ in Fig. 1d) where the *lens maker equation* would produce a bi-convex lens, while ii) if |*R*_2_|> > *R*_1_ a convex-planar lens would be formed, this feature is represented with the orange line in Fig. 1c,d corresponding to values of |*R*_2_|~ ∞. As observed from Fig. 1d, this case occurs when $\Re e\left( {n_{SPP,2} } \right) \approx 2.09$ when considering a fixed *R*_1_ = 2*λ*_0_ [i.e., when the denominator of Eq. (3) is equal to zero]. Finally, iii) if *R*_2_ > 0 (shown as the red region in Fig. 1c and values of $\Re e\left( {n_{SPP,2} } \right) > 2.09$ in Fig. 1d) a positive meniscus lens can be designed. In this realm, we can exploit this transition of *R*_2_ from negative to positive to determine the minimum $\Re e\left( {n_{SPP,2} } \right)$ capable of producing a positive meniscus lens for each *R*_1_.

In the next section, we exploit the *lens maker equation* to design plasmonic focusing devices by varying the effective refractive index $\Re e\left( {n_{SPP,2} } \right)$ to produce lenses with different shapes. Here, we focus our efforts into studying the performance of plasmonic lenses having a meniscus shape (and compare them to commonly used convex-planar designs) to demonstrate how they can be designed at optical frequencies becoming in this way an alternative lens design within the existing library of geometries for SPP focusing. The response of these plasmonic lenses will be analyzed in terms of the power enhancement, depth of focus, and transversal spatial resolution to fully study the effect that changing the shape of the plasmonic lenses will have on the produced focal spot. Finally, the broadband performance of a plasmonic meniscus lens will also be evaluated by comparing the numerically calculated *EFL* with an *EFL* directly obtained from the theoretical formulation using the *lens maker equation*.

## Results

The plasmonic lenses are then designed using Eqs. (1) and (2) following the methodology described in the previous section with an *EFL* = 2*λ*_0_ at the telecommunication wavelength of *λ*_0_ = 633 nm. We use *R*_1_ = *EFL* such that the maximum width (along the *x*-axis) of the lenses is twice the *EFL*. With this configuration, the minimum $\Re e\left( {n_{SPP,2} } \right)$ required to generate a meniscus lens is $\Re e\left( {n_{SPP,2} } \right) = 2.09$ (see the interception between the black dashed and orange lines from Fig. 1c for a visual representation of this condition).

**Figure 2:**
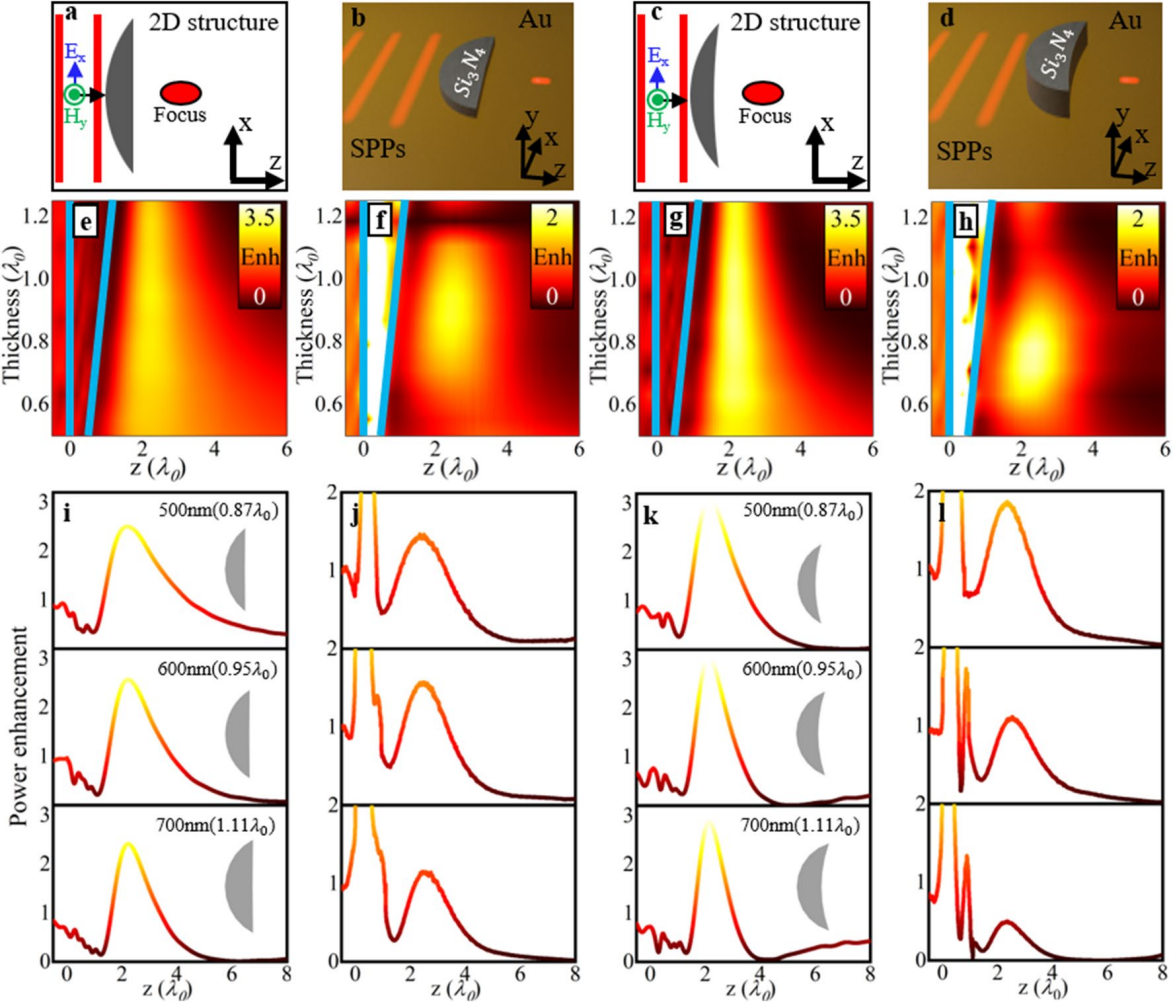
Schematic representations of the convex-planar (**a,** **b**) and the positive meniscus (**c**, **d**) lenses considered as a 2D structure illuminated with a vertically polarized plane waves (**a**, **c**) and 3D plasmonic configuration for SPP focusing (**b**, **d**). (**e**–**h**) Power enhancement on the surface of the metal (*y* = 0) at the centre of the lens (*x* = 0) for convex-planar lens (**e**) in 2D and (**f**) 3D plasmonic configurations and positive meniscus lens (**g**) in 2D and (**h**) 3D plasmonic configurations for a range of thicknesses, *t*. Note that the results shown in (**g**) has been saturated using the same scale as (**e**) to best show the improved power enhancement. Also, note the aspect ratio for the *x-* and *y*-scales in (**e**)–(**h**) are not spatially matched to better appreciate the response of the lenses when changing their thickness. (**i**–**l**) Power enhancement on the surface of the metal (*y* = 0) at the centre of the lens (*x* = 0) at specific thicknesses (0.87*λ*_0_, 0.95*λ*_0_ and 1.11*λ*_0_) when the lens is modelled as a convex-planar lens (**i**) in 2D and (**j**) 3D plasmonic configurations and positive meniscus lens (**k**) in 2D and (**l**) 3D plasmonic configurations.

As described before from Eq. (2), changing the parameter *n*_*SPP*,2_ has a significant impact on the profiles of the lens. Proximity to $\Re e\left( {n_{SPP,2} } \right) = 2.09$ (i.e., near the minimum value from where *R*_2_ changes from negative to positive), will cause $\left| {R_{2} } \right| > > R_{1}$. This will result in a profile that closely resembles a convex-planar lens. To guide the eye, this profile is schematically represented in Fig. 2a,b. On the other hand, a value of $\Re e\left( {n_{SPP,2} } \right)$ greater than the minimum of $\Re e\left( {n_{SPP,2} } \right)$ (again considering the case with *R*_1_ = *EFL* = 2*λ*_0_) will have a significantly more curved output surface as the calculated values for *R*_2_ becomes of the same order of magnitude as *R*_1_ (as shown in Fig. 2c, d). Below we will study the impact of using different output profiles on the performance of the plasmonic lens. We focus the attention on plasmonic meniscus lenses as they have the potential to achieve improved focusing performances, as it has been demonstrated for designs working at microwave/millimeter-wave frequencies^35^. Their focusing performance is compared with plasmonic convex-planar lenses. For completeness, as the plasmonics lenses are designed using the *lens maker equation* their focusing performance is compared with two-dimensional (2D) lenses (infinitely long for their out-of-plane dimension) illuminated with a plane wave, from now on we will refer to these ideal lenses as 2D lenses. Note that these 2D lenses are not plasmonic structures but correspond to ideal designs illuminated with plane waves rather than with SPPs, this will enable us to compare the 3D plasmonic lenses (full 3D designs illuminated with SPPs) and their ideal 2D counterparts. It will be shown how similar responses are obtained with the plasmonic lenses (for SPP focusing) compared to the ideal 2D lenses to demonstrate how the *lens maker equation* can be used as a good approximation for the design of plasmonic meniscus lenses.

With this in mind, two values of complex *n*_*SPP*,2_ are chosen (due to the dispersive nature of Au): first we used *n*_*SPP*,2_ = 2.09 + i0.092. From Eq. (1), this value corresponds to a height of the Si_3_N_4_ dielectric of *l*_*y*_ = 65 nm. Note that this value of $\Re e\left( {n_{SPP,2} } \right)$ is close to the limit where *R*_2_ is infinite from Fig. 1c, d (orange line). As explained before, the profile of a plasmonic lens with a value of *R*_2_ = ∞ will correspond to a convex-planar lens [Eq. (3)]. In our case, *R*_2_ is positive and close to (but not exactly) infinite, i.e., strictly speaking, a quasi-convex-planar design. However, as *R*_2_ >> *R*_1_ from now on we call this design as a convex-planar plasmonic lens. For the second design, we used *n*_*SPP*,2_ = 2.43 + i0.075, a value that falls well above the *R*_2_ = ∞ line from Fig. 1c, d (orange line), i.e., a profile corresponding to that of a positive plasmonic meniscus lens (convex-concave profile). It is important to note that, as shown in Fig. 1c, d, such meniscus geometries can be achieved when *n*_*SPP*,2_ is increased. However, the maximum value of $\Re e\left( {n_{SPP,2} } \right)$ depends on the materials used for the IIM region [as described in Eq. (1)]. For the materials used in this work, $\Re e\left( {n_{SPP,2} } \right)$ starts to saturate to its maximum of ~ 2.47 as *l*_*y*_ approaches 200 nm (not shown). In this context, we can choose the Si_3_N_4_ dielectric height to be *l*_*y*_ = 126 nm so we work below saturation. With this parameter *l*_*y*_ = 126 nm, the $\Re e\left( {n_{SPP,2} } \right) = {2}.{43}$^34^. Based on this, in the following designs we make use of these two extreme values of *n*_*SPP*,2_ (*n*_*SPP*,2_ = 2.09 + i0.092 and *n*_*SPP*,2_ = 2.43 + i0.075) along with Eq. (2) to design the convex-planar and meniscus lenses by calculating the value of the output surface’s radius of curvature, *R*_2_, for each case. For the sake of completeness, we will also vary the thickness *t* of the plasmonic lenses from 0.5*λ*_0_ to 1.25*λ*_0_ to study their focusing properties more comprehensively. Note that the full *lens maker equation* described in Eqs. (2) and (3) will be implemented for each value of *t*.

### Power enhancement at the focus

The plasmonic convex-planar and meniscus lenses are numerically studied using the frequency domain solver of the commercial software COMSOL Multiphysics following the same setup as in Refs.^5,17^ (see Methods section for further details). To fully compare the performance of the plasmonic lenses, we consider two situations: (i) a 3D scenario where a 3D plasmonic lens is illuminated by SPPs propagating along the *z*-axis and (ii) a 2D scenario where a 2D meniscus lens is illuminated by a plane wave (polarized along *y*) and propagating along the *z*-axis. In the former 3D case, the effective refractive index of the SPPs propagating in the air-Si_3_N_4_-Au region (i.e., the plasmonic lens) is *n*_*SPP*,2_ = 2.09 + i0.092 and *n*_*SPP*,2_ = 2.43 + i0.075 for the convex-planar and meniscus designs, respectively, with the background medium (air–Au region) having an effective refractive index of *n*_*SPP*,1_ = 1.045 + i0.005. These designs are schematically shown in Fig. 2b, d. For the 2D designs (shown in Fig. 2a, c) the refractive index of the 2D lenses and background medium is chosen to be equal to *n*_*SPP*,2_ and *n*_*SPP*,1_, respectively, i.e. the same values used for the 3D plasmonic lenses from Fig. 2b, d, in order to compare the 3D plasmonic lenses with the ideal designs illuminated with a plane wave.

With this setup, the numerical results of the power enhancement (defined as the ratio of the power distribution with and without the plasmonic lens) along the propagation *z*-axis calculated on the surface of the metal (*y* = 0) at the center of the lens (*x* = 0) are shown in Fig. 2e–h. Here we evaluate the ideal 2D lenses (Fig. 2e, g) and the 3D plasmonic lenses (Fig. 2f, h) considering the two values of *n*_*SPP*,2_:*n*_*SPP*,2_ 2.09 + i0.092, convex-planar (Fig. 2e, f) and *n*_*SPP*,2_ = 2.43 + i0.075, meniscus (Fig. 2g, h). A range of thicknesses from *t* = 0.5*λ*_0_ (316 nm) to *t* = 1.25*λ*_0_ (791 nm) are considered for all the designs. Note that, according to Eq. (3), the value of *R*_2_ is recalculated for every value of thickness (*t*). The input (*z* = 0) and output surfaces (*z* = *t*), at *x* = *y* = 0, for all the lenses are represented in Fig. 2e–h as blue lines to guide the eye.

As shown in Fig. 2e–h, a clear focus is obtained for all the designs. From Fig. 2e, g, the 2D lenses produce a focus regardless of the lens thickness. However, for thinner lenses with *t* < 0.75*λ*_0_ the focus becomes elongated along the propagation axis and the power enhancement is reduced. This performance may be due to the lens being too thin and most of the focus is produced by diffraction from the edges of the lens. The 3D plasmonic cases shown in Fig. 2f, h also generate a focus for values of 0.6*λ*_0_ < *t* < 1.1*λ*_0_. Outside this region no clear focusing of SPPs is achieved as a result of the lens either being too thin to allow SPPs to be redirected to the focal point (similar to the 2D case shown in Fig. 2e, g) or too thick so the SPPs suffer from greater losses from the extended propagation length inside the lens, again affecting the formation of focus.

However, by comparing the 2D convex-planar and meniscus lenses with their 3D plasmonic versions for SPP focusing, it can be seen how both meniscus designs have an improved enhancement at the focal point compared to the convex-planar designs. For instance, for the best performing cases of each geometry, a value of *t* = 600 nm (~ 0.95λ_0_) when considering a convex-planar profile will result in the maximum power enhancement being 2.57 and 1.57 for the 2D lenses and 3D plasmonic designs, respectively, while for *t* = 500 nm (~ 0.79λ_0_) when using meniscus geometries, it is 3.14 and 1.83, respectively. This response is due to the pronounced curvature of the output surface of the meniscus designs which allows redirecting SPPs towards the focal spot for more output angles. Moreover, it is important to note how the focusing performance is improved for the 2D/3D meniscus lenses even when their thickness is considerably smaller (compact) than the convex-planar designs. Interestingly, both the 2D and 3D configurations are in agreement with meniscus lenses at microwave frequencies as reported in different scenarios such as in antennas^35^ and field correctors^29^, emonstrating how classical optics techniques for meniscus lenses can be also applied to plasmonic devices. Next, we evaluate if the position of the focus agrees with the designed value (*EFL* = 2λ_0_ in our case).

### Effective focal length and depth of focus

**Figure 3:**
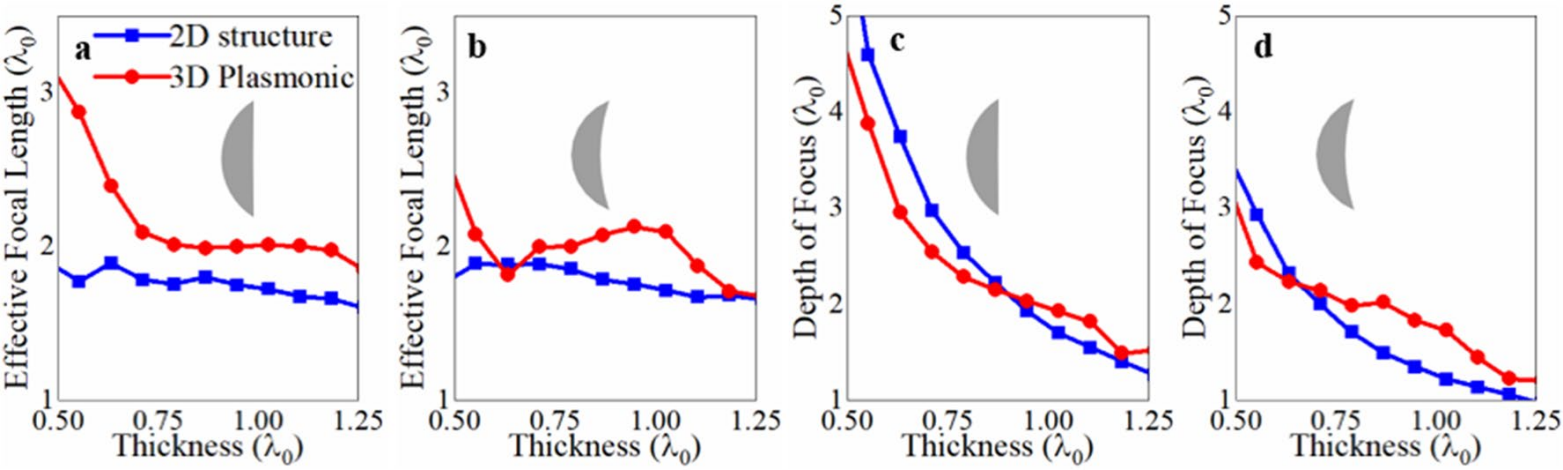
Effective focal length for the 2D lenses illuminated with a plane wave (blue) and 3D plasmonic designs (red) considering (**a**) convex-planar and (**b**) meniscus geometries. Depth of focus for the 2D lenses illuminated with a plane wave (blue) and 3D plasmonic (red) designs having (**c**) convex-planar and (**d**) meniscus output surfaces.

We extracted the *EFL* from Fig. 2e–h and the results are shown in Fig. 3a, b for both 2D structures illuminated with a plane wave (red) and the 3D plasmonic versions (blue). We consider both profiles under study: convex-planar (Fig. 3a) and meniscus (Fig. 3b). By comparing the results shown in Fig. 3a, b, one can notice how similar values are obtained with the ideal 2D versions illuminated with a plane wave compared to the plasmonic structures for SPP focusing. For instance, when *t* = 600 nm (~ 0.95λ_0_), the *EFL* for the convex-planar lens (Fig. 3a) as a 2D structure and as a 3D plasmonic structure is 1.75*λ*_0_ and 1.97*λ*_0_, respectively, while the meniscus lens for the same 2D and 3D configurations is *EFL*s of 1.75*λ*_0_ and 2.13*λ*_0_, respectively.

For completeness, we show in Fig. 3c, d, the depth of focus (DoF, defined as the distance at which the power enhancement at the focal length has decayed to half of its maximum along the direction of propagation, *z*^36^) for the designs shown in Fig. 2. As observed from these results, as the thickness, *t*, of the lens is increased there is a decrease in the DoF, as thicker lenses better redirect SPPs from positions further away along the *x*-axis (larger output angles) towards the desired focal point, resulting in a reduced focal spot in the direction of propagation. Moreover, by comparing the 2D structures that are illuminated with a plane wave (blue curves) and the 3D plasmonic structure (red curves) from Fig. 3c, d, one can see that the DoF follows a similar trend: for values of *t* < 0.75*λ*_0_ the generated focal spots have a larger DoF before decreasing significantly for *t* ≈ 1*λ*_0_. For the 2D and 3D plasmonic convex-planar designs, the DoF varies from ~ 5.3*λ*_0_ to ~ 1.9*λ*_0_ and from ~ 4.5*λ*_0_ to ~ 2.03*λ*_0_ when using values of *t* between 0.5*λ*_0_ and 0.95*λ*_0_, respectively. For the meniscus designs, the DoF varies from ~ 3.5*λ*_0_ to ~ 1.3*λ*_0_ and from ~ 3*λ*_0_ to ~ 1.82*λ*_0_ for the 3D plasmonic lens and 2D design, respectively, for the same range of *t* (0.5*λ*_0_ and 0.95*λ*_0_). These results demonstrate that the overall performance of the 3D plasmonic lenses agree well with those expected from the ideal 2D lenses illuminated with a plane wave.

It is important to highlight how the performance of the focus in terms of DoF is improved for the meniscus lenses compared with the convex-planar designs using both plane wave illumination (2D lenses) and SPP-based plasmonic structures (3D lenses): for instance, for 3D plasmonic designs with a thickness of *t* = 700 nm (1.11*λ*_0_) the convex-planar lens has a DoF = 1.92*λ*_0_ and for the meniscus lens, it is DoF = 1.72*λ*_0_. The same response is observed for the 2D lenses where a DoF of 1.54*λ*_0_ and 1.13*λ*_0_ is achieved for the convex-planar and meniscus geometries, respectively. These results show how the meniscus lens can produce smaller focal spots along the propagation direction. Note that these results are as expected because of the curved output surface of the meniscus lenses (as described above), a performance known for such lenses in free space^37^ and corroborated in this work with the 2D ideal structures. Hence, this work demonstrates how similar performances can be achieved when applying such classical optics techniques in plasmonic structures for SPP focusing using meniscus geometries.

### Transversal resolution

**Figure 4:**
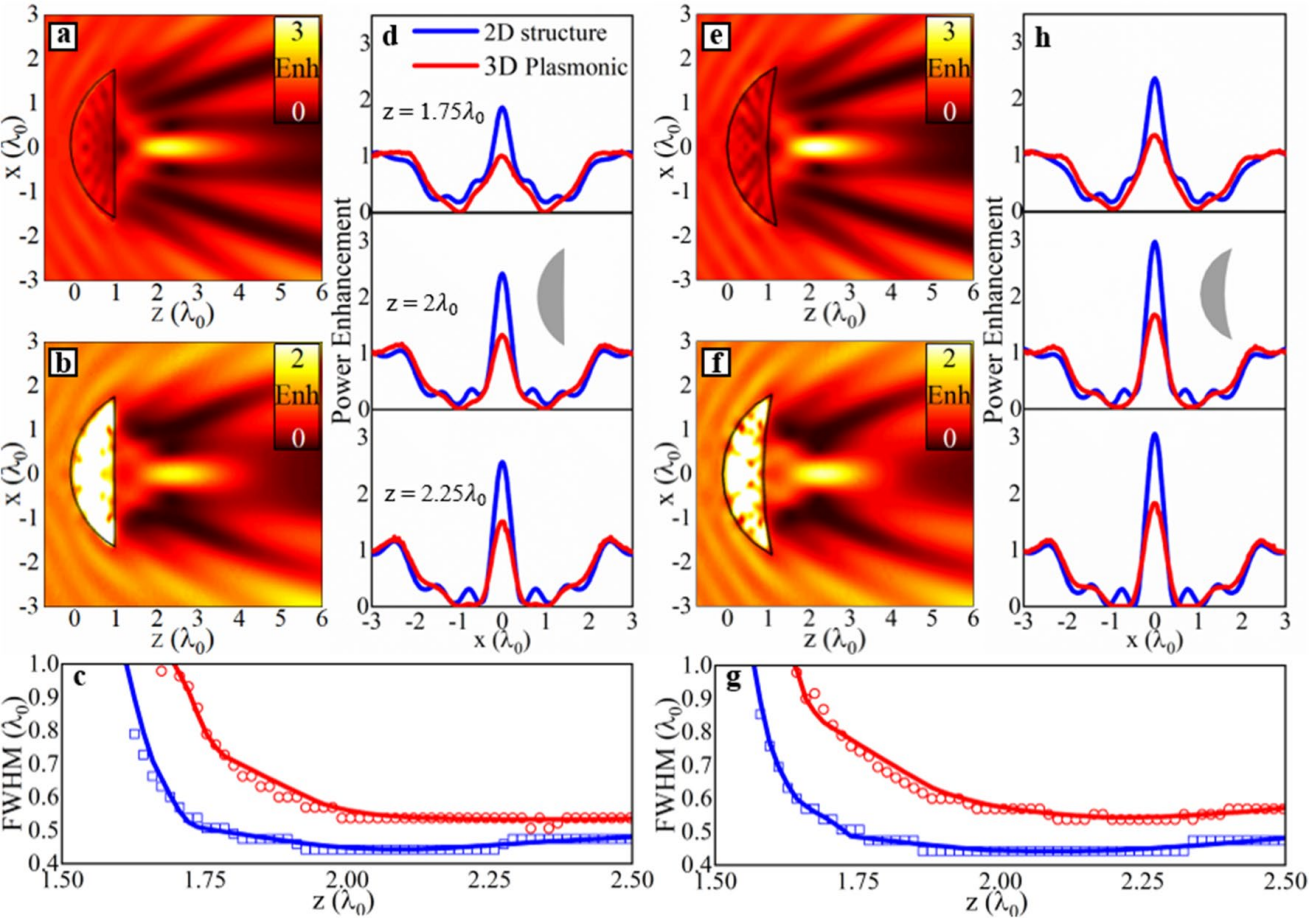
Power enhancement on the *xz*-plane for (**a**, **b**) convex-planar lens with a thickness of *t* = 600 nm (0.95*λ*_0_) and (**e**, **f**) meniscus lenses of thickness *t* = 500 nm (~ 0.79*λ*_0_) considering 2D designs illuminated with a plane wave (**a**, **e**) and 3D plasmonic structures (**b**, **f**). Power enhancement for the (**d**) convex planar and (**h**) meniscus lenses along the transversal direction at three different positions in the direction of propagation, *z* = 1.75*λ*_0_, *z* = 2*λ*_0_ and *z* = 2.25*λ*_0_. Insets in panels (**d**) and (**h**) schematically represent the profile of the lenses. *FWHM* at different positions along the propagation axis for a (c) convex-planar lens and (**g**) meniscus lens. The results obtained directly from the numerical simulations and the resulting trend are presented as symbols and solid lines, respectively.

Another important parameter of focusing devices is the resolution of the focus on the focal plane (*xz*-plane in our case) in terms of the Full-Width at Half-Maximum (*FWHM*, defined as the full width of the peak at half of the maximum power along the transversal direction^18^). To evaluate the transversal resolution, we can select a single thickness from the lens designs shown in Fig. 2. Following the results from the previous section, here we select *t* = 600 nm (0.95*λ*_0_) for the convex-planar geometry and *t* = 500 nm (0.79*λ*_0_) for the meniscus profile, as they correspond to the designs with maximum power enhancement at the resulting *EFL*s. Moreover, note that designs have very simiar *EFLs* (1.99905 and 1.99916, respectively) when using such values of *t*. Hence, by using *t* = 600 nm and *t* = 500 nm for the convex-planar and meniscus profiles, respectively, we ensure a fair comparisson of the transversal resolution as smaller/larger *EFLs* would lead to narrow/wider focal spots, as expected^38^, generating misleading conclusions. With this configuration, the numerical results of the power enhancement on the *xz*-plane at *y* = 0 are shown in Fig. 4a, b for the convex-planar design (with *n*_*SPP*,2_ = 2.09 + i0.092) considering the 2D structure and 3D plasmonic lens, respectively. The results of the power enhancement for the plasmonic meniscus design with *n*_*SPP*,2_ = 2.43 + i0.075 are also shown in Fig. 4e, f for the same 2D and 3D configurations, respectively. As it can be observed, the 2D lenses and 3D plasmonic lenses produce very similar focal spots. The main difference is the power enhancement at the focus as the plasmonic lenses have been modeled as realistic 3D designs illuminated by SPPs rather than the 2D structures illuminated with a plane wave. This results in increased losses and hence a drop in power enhancement at the focus for the 3D devices. Also note how scattering occurs in all of the designs due to the abrupt change of refractive index between the lens and the surrounding medium. This could be minimized by exploiting gradient index designs^27,39^ where multiple layers with different materials could be added at the back of the lens to reduce the need of using a single step of refractive index between the IM and IIM regions (background and dielectric) for the 3D plasmonic lenses (2D lenses).

The final stage in evaluating the performance of the convex-planar and meniscus lenses is to compare their spatial resolution along the transverse axis. This can be carried out by obtaining the *FWHM* along the *x*-direction for all the configurations shown in Fig. 4a, b, e, f. The results of power enhancement along the transverse axis for three different positions along the direction of propagation: namely *z* = 1.75*λ*_0_, *z* = 2*λ*_0_ and *z* = 2.25*λ*_0_ are shown in Fig. 4d, h, considering 2D and plasmonic 3D convex-planar and meniscus lenses, respectively. By comparing the power enhancement distribution at each *z*-position, it is clear that, in general, the power enhancement is increased for both 2D and 3D meniscus profiles, compared to the convex-planar cases, in agreement with the discussion presented above. To further evaluate their response, the *FWHM* at positions along *z* ranging from 1.5*λ*_0_ to 2.5*λ*_0_ were calculated and the results are shown in Fig. 4c, g for both the 2D/3D plasmonic convex-planar and meniscus designs, respectively. As observed, the spatial resolution along the transversal direction is similar for both designs. For instance, the *FWHM* at the focal point of the meniscus lens (Fig. 4c) is 0.44*λ*_0_ and 0.54*λ*_0_ for the 2D structure and the 3D plasmonic structure, respectively, while the convex-planar 2D lens and 3D plasmonic lens (Fig. 4g) have the same *FWHM* values of 0.44*λ*_0_ and 0.54*λ*_0_, respectively. Based on these results, one can conclude that using plasmonic meniscus lenses (with a concave output surface) will have a significant impact upon the propagation of SPPs resulting in an overall improvement of the focal spot in terms of power enhancement, the depth of focus with similar transversal resolution when compared to a convex-planar lens. We would also like to note that, while here we focused our attention on the design and study of plasmonic meniscus lenses for SPP focusing, our technique could also be translated into other technologies such as metasurface-based lenses for free-space focusing where they can be designed to emulate the phase profile of a meniscus lens via gradient index techniques^15,40^.

### Broadband response

Finally, let us evaluate the broadband response of a plasmonic meniscus lens designed to work at the telecommunication wavelength *λ*_0_ = 633 nm. To do this we used the parameters required to satisfy Eqs. (1) and (2) to design a meniscus profile with an *EFL* = 2*λ*_0_ = 1266 nm. Here we make use of the same designs described in the previous section with *n*_*SPP*,1_ = 1.045 + i0.005 for region 1 (air-Au) and *n*_*SPP*,2_ = 2.43 + i0.075 for region 2 (air-Si_3_N_4_-Au) using a dielectric height of *l*_*y*_ = 126 nm. These values were then used to generate a value for the ratio between *n*_*SPP*,2_ and *n*_*SPP*,1_, Φ, to use in Eq. (2). Then using the method described previously in Eq. (3) with *R*_1_ = 1266 nm, *t* = 500 nm, and an *EFL* = 1266 nm a value of *R*_2_ = 4046 nm was calculated. These parameters were then used to generate a profile for a meniscus lens which was evaluated within the spectral range of 550–750 nm.

As it is known, changing the wavelength will have a direct impact on both the IM and IIM working regions where the SPPs travel as the properties of both Au and Si_3_N_4_ will change due to their dispersive nature. Hence, the effective refractive index of regions 1 and 2 (*n*_*SPP*,1,2_), see insets of Fig. 1a, will also change according to Eq. (1). Therefore, using the parameters from the plasmonic meniscus lens we have designed in the previous sections, we can then use Eq. (2) with the newly calculated *n*_*SPP*,1,2_ to determine the shift which would occur to the *EFL*. This is an example of the chromatic aberrations which will occur in lenses based on the *lens maker equation* in a dispersive setting, as those designed in this work. These analytically calculated values of the *EFL* can then be compared to the simulated results for the plasmonics lens to fully study its performance.

**Figure 5:**
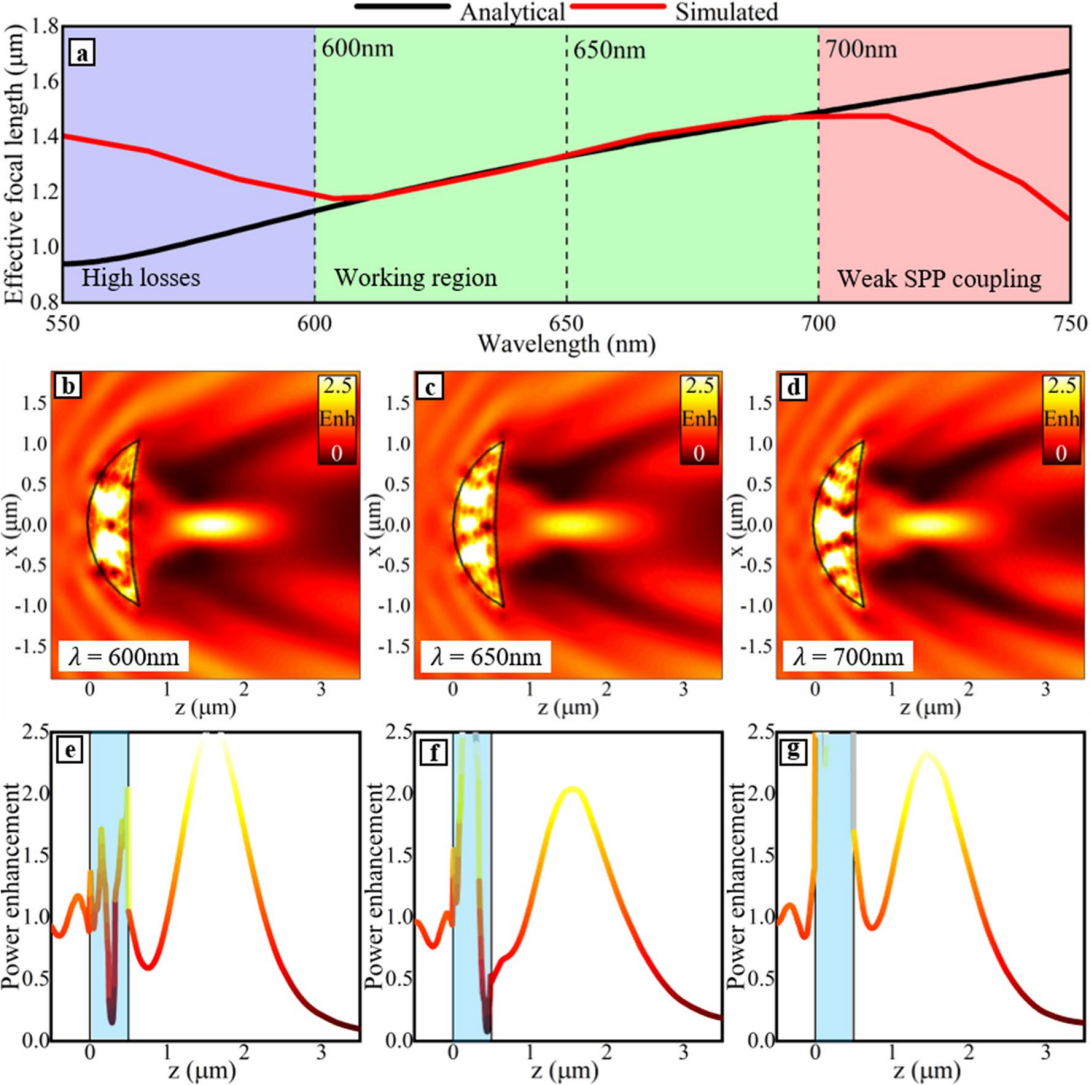
(**a**) Theoretical (black) and numerical (red) results of the *EFL* as a function of the incident illumination wavelength for the 3D plasmonic meniscus lens discussed in Fig. 4f designed at *R*_1_ = 1266 nm, *R*_2_ = 4046 nm, *t* = 500 nm, and an *EFL* = 1266 nm. The blue region (λ between 550 and 600 nm) represents a spectral band where the meniscus lens suffers from high losses, the green region (λ between 600 and 700 nm) represents where the *lens maker equation* functions as expected*,* and the red region (λ between 700 and 750 nm) represents where the lens suffers from a weak coupling of SPPs. Power enhancement on the *xz*-plane at the surface of the metal (*y* = 0) for the case described previously for a wavelength of (**b**) 600 nm (**c**) 650 nm and (**d**) 700 nm. Power enhancement on the surface of the metal (*y* = 0) for a wavelength of (**e**) λ = 600 nm, (**f**) λ = 650 nm and (**g**) λ = 700 nm along the *z*-direction at the centre of the lens (*x* = 0). The blue boxes show the position of the lens along the *z*-direction at the centre of the lens (*x* = 0). The scale bars seen in (**b**)–(**d**) also refer to colouring in (**e**)–(**g**).

With this setup, the broadband response of the plasmonic meniscus lens is presented in Fig. 5a where we show the analytically calculated *EFL* (black line) along with the values obtained via numerical simulations (red line). For wavelengths between 600 and 700 nm, we can see how both theoretical and numerical results are in excellent agreement. We call this the “working region” corresponding to the spectral range where the plasmonic meniscus lens behaves as predicted by the *lens maker equation* [Eq. (2)]. Outside of this range, the results deviate. Below 600 nm, between 550 and 600 nm, marked in Fig. 5a as the blue region, the theoretical and numerical results differ mainly due to the increased losses experienced by SPPs when reducing the working wavelength, as we have shown in^5,34^. Such a lossy scenario is not present in the *lens maker equation* as it considers lossless lenses, hence producing a deviation of results. This causes the focus of the simulated 3D plasmonic meniscus lens to shift away from the theoretical values as the *lens maker equation* does not consider losses. Therefore, as the effect of losses increases as does the deviation between the analytically calculated values and the numerically simulated *EFL*. To address these differences, a further potential modification to the *lens maker equation* could be to consider the effect of losses of SPPs in the IM and IIM regions directly in the design, a study that will be developed in the future. On the other hand, for wavelengths above 700 nm (marked in Fig. 5a by the red region) the numerically calculated *EFL* has smaller values than the analytical results. This effect occurs due to the SPPs being less confined to the metal surface when increasing the operational wavelength, as has been shown theoretically in^1,14,41–43^, causing a deviation of results as the theoretical calculations consider a perfect coupling of SPPs which does not occur for longer wavelengths, as expected.

For completeness, we show in Fig. 5b–d, the power enhancement on the *xz*-surface of the metal (*y* = 0) of the plasmonic meniscus lens at three different wavelengths (*λ* = 600 nm, 650 nm, 700 nm, vertical dashed lines in Fig. 5a). Moreover, we extracted the power enhancement on the surface of the metal (*y* = 0) at the center of the lens (*x* = 0) at the same wavelengths and the results are shown in Fig. 5e–g, respectively. From these results, we can corroborate the effect of the operational wavelength on the performance of the plasmonic meniscus lens. For *λ* = 600 nm the focus was found at an *EFL* = 1.19 μm for the numerical simulations, a slight deviation compared to the analytically calculated value of 1.13 μm. For a wavelength of *λ* = 650 nm, the numerical results provided an *EFL* of 1.33 μm which was the same as the analytically calculated *EFL*. Finally, when *λ* = 700 nm, the simulated focus appeared at 1.47 μm while a value of 1.49 μm was obtained theoretically. These results demonstrate how the plasmonic meniscus lens can work as expected within the spectral region of 600–700 nm. From these results, we can conclude that the *lens maker equation* is effective for the design of plasmonic focusing devices such as plasmonic meniscus lenses when working within spectral optical ranges where the SPPs are strongly confined to the surface of the metal and do not suffer too greatly from the intrinsic losses.

Finally, an in-depth investigation into the robustness of the proposed plasmonic meniscus lenses is presented in the supplementary materials where four different studies similar to the one shown in Fig. 5 are presented**.** First, a study of the effect of potential experimental errors which may occur to the height of the dielectric layer is presented in Section 1 of the supplementary materials. In those results, it is shown how a change of height can indeed modify the effective refractive index of SPPs traveling inside the IIM region (*n*_*SPP*,2_), as expected, which generates a shift of position of the focus. However, it is shown how the new *EFL* can be analytically predicted using Eqs. (1)–(2). In a second study, the effect of changing the lens thickness (*t*) on the *EFL* is presented in Section 2 of the supplementary material. These results again demonstrate how the focal spot shifts its position when modifying the *t* due to potential experimental errors while such displacement can be reliably predicted theoretically using Eq. (2).

A further study into the potential effects of errors to the focusing devices is presented in Section 3 of the supplementary material where potential errors due to nanofabrication limitations are studied^44–48^. Here, it is considered that the edges of the plasmonic meniscus lenses are not sharp but rounded due to fabrication constraints. In this context, the edges of the plasmonic meniscus lenses were designed to be rounded with only features larger than 100 nm (non-sharp corners). As discussed in Section 2 from the supplementary materials, the effect of rounded edges is minimal with a slight decline in power enhancement at the focal spot compared to the ideal plasmonic meniscus lenses having ideal sharp edges.

For the sake of completeness, a study of the focusing performance of the proposed plasmonic meniscus lenses under off-axis oblique illumination is presented in Section 4 of the supplementary material. Here, a plasmonic meniscus lens was rotated on the *xz*-plane with angles ranging from 0° to 30° in steps of 3°. As shown in Fig. S4, a rotation of the plasmonic meniscus lens generates a slight change of the position of the focus (as expected). However, it is shown that the lens can be rotated up to 15° without having an important impact (reduction) of the power enhancement at the focal spot.

## Discussion

In this work, we applied the *lens maker equation* as a technique known in classical optics to design 3D plasmonic meniscus lenses for SPP focusing at optical frequencies. The designs have been compared with their ideal 2D configurations considering plane wave illumination demonstrating a good agreement between them. It has been shown that plasmonic positive meniscus lenses offer an improvement in the performance of the focal spot in terms of reduced depth of focus and increased power enhancement compared to commonly used convex-planar geometries. We have also shown the broadband capabilities of a plasmonic meniscus lens being able to produce a focus in agreement with an analytically calculated *EFL* in a 100 nm range from 600 to 700 nm. The results here presented may open alternative designs for manipulations of SPPs at optical frequencies and may find applications in sensing with future investigations into their potential to produce optical forces for use as optical tweezers.

## Methods

All the numerical simulations were performed using the frequency-domain solver of the commercial software COMSOL Multiphysics. for the 2D structures, a rectangular box of dimensions 14.7*λ*_0_ × 49.0*λ*_0_ was implemented. The incident plane wave was applied from the back/left boundary of the simulation box via a scattering boundary condition with an out-of-plane magnetic field (electric field polarized along the *y*-axis). Scattering boundary conditions were also implemented on the front/right boundaries of the simulation box. Finally, a triangular mesh was implemented with a minimum and maximum size of 2.93 × 10^−4^*λ*_0_ and 0.30*λ*_0_ respectively, to ensure accurate results. For the 3D simulations, a metallic cuboid slab of dimensions 18.4*λ*_0_ × 9.8*λ*_0_ × 0.4*λ*_0_ was implemented. Then 4 slits of 0.18*λ*_0_ spanning the width of the slab were periodically placed every 0.61*λ*_0_ in the direction of propagation. An incident field was applied at the bottom of the slits via a scattering boundary condition. Scattering boundary conditions were also implemented on the bottom, left, and right boundaries of the simulation box to avoid undesirable reflections. Finally, a triangular mesh was implemented with a minimum and maximum size of 2.22 × 10^−2^*λ*_0_ and 0.47*λ*_0_, respectively, to ensure accurate results. With this configuration the SPPs were excited at the interface of the metallic bock and a dielectric, polarized along *x* and propagating along the *z*-axis.

## Supplementary Information

Supplementary Information.
