# Supplementary material for: Plasmonic meniscus lenses
Source: Sci Rep. 2022 Jan 18;12:894. doi: 10.1038/s41598-022-04954-0 (PMC8766487; doi:10.1038/s41598-022-04954-0)
Supplement: Supplementary file 1 — Supplementary Information. [file 41598_2022_4954_MOESM1_ESM.pdf]

Supplementary information

Plasmonic Meniscus Lens

Joseph Arnold Riley[1,2], Noel Healy[1] and Victor Pacheco-Peña[1]*

[1] School of Mathematics, Statistics and Physics, Newcastle University, Newcastle Upon Tyne, NE1 7RU, United Kingdom
[2]School of Engineering, Newcastle University, Newcastle Upon Tyne, NE1 7RU, United Kingdom
*email: victor.pacheco-pena@newcastle.ac.uk

1. **Effect of potential fabrication errors of the height of the Si_3N_4 layer on the generated effective focal length (EFL).**
2. **Effect of potential fabrication errors of the lens thickness (*t*) on the generated EFL.**
3. **Effect of potential fabrication errors causing vanishing edges.**
4. **Effect of oblique illumination on a plasmonic meniscus lens.**

1. Effect of potential fabrication errors of the height of the Si_3N_4 layer on the generated effective focal length (EFL).

Here we consider the plasmonic meniscus lens discussed in Figure 5 from the main text with dimensions R_1= 1266nm ($2\lambda_0$), R_2 = 4046nm ($6.39\lambda_0$), t = 500nm ($0.79\lambda_0$), designed to have an *EFL* at 1266nm ($2\lambda_0$) and a height of the Si_3N_4 layer of 126nm with an operational wavelength of 633nm. The simulation and analytical (using Equations 1 and 2 from the main text) *EFL* results when changing the height of the Si_3N_4 are shown in Figure S1. As observed, there is a good agreement between the results demonstrating the validity of the proposed design process. For completeness, examples of the power distribution on the *xz*-plane at y = 0 for different heights of the Si_3N_4 layer are shown in the same figure, demonstrating that a focus is still produced even if the height of the dielectric has some experimental errors.

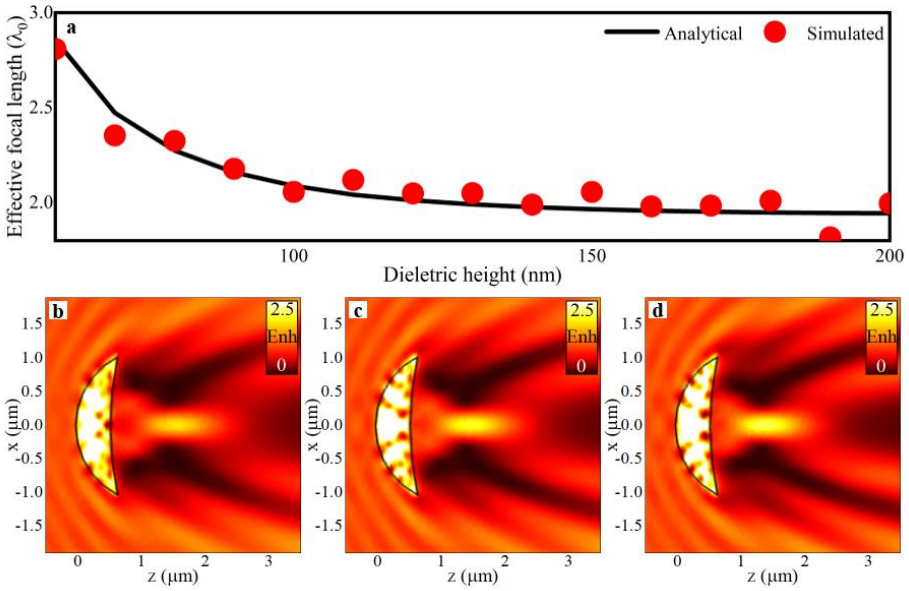

Figure S1. (a) Analytically calculated (black) and numerical (red) results of the *EFL* as a function of the height of the dielectric used for the 3D plasmonic meniscus lens discussed in Figure 5 designed at R_1= 1266nm ($2\lambda_0$), R_2 = 4046nm ($6.39\lambda_0$), t = 500nm ($0.79\lambda_0$), designed to have an EFL at 1266nm ($2\lambda_0$) and a height of the Si_3N_4 layer of 126nm. Power enhancement on the *xz*-plane at the surface of the metal (y = 0) for the case described previously for a dielectric height of (b) 100nm (c) 150nm and (d) 200nm.

2. Effect of potential fabrication errors of the lens thickness (t) on the generated EFL.

Here we consider the plasmonic meniscus lens discussed in Figure 5 from the main text designed with dimensions R_1 = 1266nm ($2\lambda_0$), R_2 = 4046nm ($6.39\lambda_0$), t = 500nm ($0.79\lambda_0$), to produce an *EFL* at 1266nm ($2\lambda_0$) with an operational wavelength of 633nm. The thickness of the lens at x = 0 is then varied using fixed values of R_1 and R_2 as the designed lens. The simulation and analytical (using Equations 1 and 2 from the main text) results of the *EFL* when the t of the plasmonic meniscus lens is changed from 300nm ($0.47\lambda_0$) to 800nm ($1.26\lambda_0$) with a step of 50nm ($0.08\lambda_0$) are shown in Figure S2a. As observed, there is a good agreement between the results for the designs where the generated power enhancement at the focal spot is greater than 1 (similar to the performance shown in Figure 2 from the main text), demonstrating the validity of the proposed design process when a focus is produced. For completeness, examples of the power enhancement on the *xz*-plane at y = 0 for t = 500nm, 600nm, and 700nm are shown in Figure S2b-d, respectively. These results demonstrate that as long as the errors are not too great as to make the lens too thick or thin and remain in the region t ~ 0.6 - $1\lambda_0$, see Figure 2 from the main text, a focus with a higher than 1 power enhancement will be produced with a focal position that can be analytically predicted using the adapted lens maker equation from Equation 2 of the main text, demonstrating the robustness of the lens to overcome some potential experimental errors.

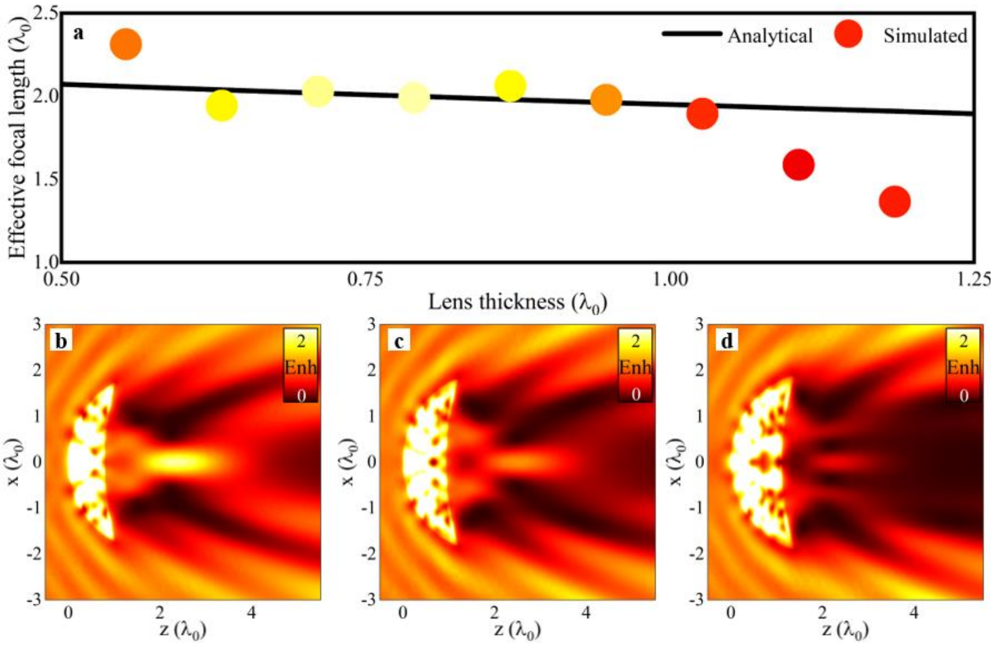

Figure S2. (a) Analytically calculated (black) and numerical (colour scaled) results of the EFL as a function of the thickness of the plasmonic meniscus lens at x = 0, designed with R_1= 1266nm ($2\lambda_0$), R_2 = 4046nm ($6.39\lambda_0$), t = 500nm ($0.79\lambda_0$), with an *EFL* = 1266nm ($2\lambda_0$) and a height of the Si_3N_4 dielectric of 126nm (as discussed in Figure 5 from the main text). The colour of the data points represents the power enhancement at each lens thickness using the same scale bars as seen in (b-d). Power enhancement on the *xz*-plane at the surface of the metal (y = 0) for the plasmonic meniscus lenses from panel (a) with (b) t = 500nm (c) t = 600nm and (d) t = 700nm

3. Effect of potential fabrication errors causing vanishing edges.

Here we consider the effect that current constraints of nanofabrication methods would have on the designs of plasmonic meniscus lens we have shown in the main text. Due to the current limitations of nanofabrication techniques the smallest features possible to reliably fabricate are in the region of tens of nanometers[44-48]. In this context, it could be possible that the sharp edges of the plasmonic meniscus lenses here proposed may 'vanish', introducing potential errors in the final plasmonic design. In this Section, we explore the effect that these fabrication errors may have on the performance of plasmonic meniscus lenses. To do this we designed several plasmonic Si_3N_4 meniscus lenses using the adapted *lens maker equation* from the main manuscript to have an *EFL* of 1266nm ($2\lambda_0$) working at an operational wavelength of 633nm. With this configuration, the effective refractive index of the dielectric-dielectric-metal region is n_{eff} = 2.43 using Equation 1 from the main text. Moreover, we consider R_1 = 1266nm ($2\lambda_0$) and the thickness, *t*, was then varied so R_2 could be calculated using Equation 2 and 3 from the main text. To account for the potential imperfect edges of the plasmonic meniscus lenses, we replaced the sharp well-defined edges used in the main text of the manuscript with rounded corners so there were no features smaller than 100nm, i.e., working above the lowest tolerances of nanofabrication.

With this configuration, we show examples of the power enhancement on the *xz*-plane at *y* = 0 for different lens thicknesses (400nm - 700nm, step of 100nm) with rounded corners in Figure S3a-d. As observed, clear foci are produced by all the plasmonic meniscus lenses even when their edges are not sharp. However, note that having the lens too thick lowers the enhancement, as expected from Figure 2 due to the increasing losses of SPPs traveling within the dielectric-dielectric-metal region. To better analyse these results, we extracted the power distribution along the *z*-axis at the metal-dielectric interface (*y* = 0) and centre of the lens (*x* = 0) for the plasmonic lenses from Figure S3a-d. The results are plotted in Figure S3e-f, respectively, as red lines along with the power distribution along the *z*-axis considering the same designs but using the ideal sharp edges (black plots). From these results, one can observe that there is a good agreement between both ideal sharp and rounded edges, with only a slight reduction of the power enhancement for cases with smaller values of *t* (given that the curvature of the rounded edges becomes comparable to the thickness *t*). These results demonstrate the potential robustness of the proposed plasmonic meniscus lenses to potential fabrication errors.

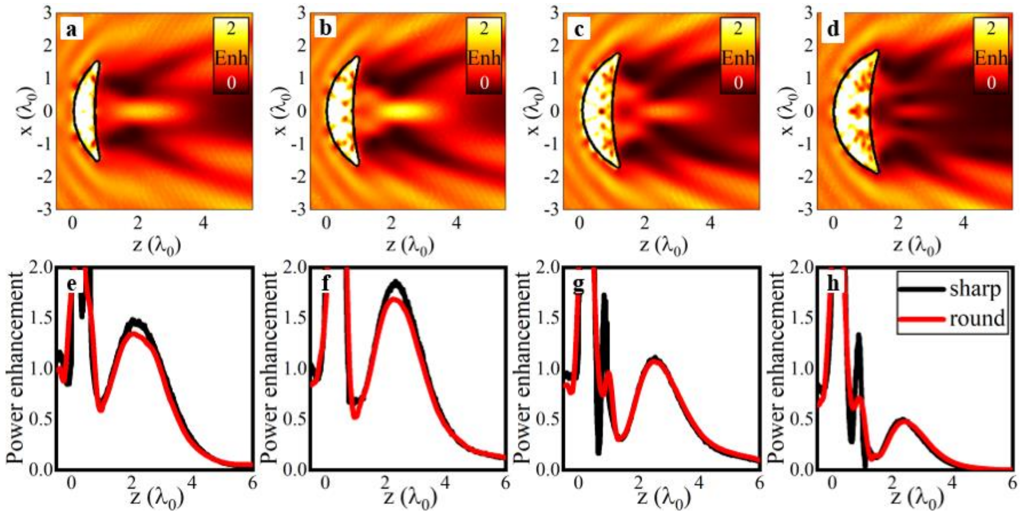

Figure S3. Power enhancement on the *xz*-plane at the surface of the metal (y = 0) for four plasmonic meniscus lenses with a thickness (a) t = 400nm, (b) t = 500nm, (c) t = 600nm and (d) t = 700nm with the edges rounded so there are no features less than 100nm thick. (e-f) Power enhancement on the surface of the metal (y = 0) at the centre of a lens (x = 0) with sharp corners (black) and for rounded edges (red) for plasmonic meniscus lenses from panels (a-d), respectively.

4. Effect of oblique illumination on a plasmonic meniscus lens.

Here we consider the plasmonic meniscus lens discussed in Figure 5 from the main text [R_1= 1266nm ($2\lambda_0$), R_2 = 4046nm ($6.39\lambda_0$), t = 500nm ($0.79\lambda_0$), designed to have an *EFL* at 1266nm ($2\lambda_0$) with an operational wavelength of 633nm] and evaluate its focusing performance under oblique incidence. The plasmonic lens was placed at the original position as shown in Figure 5 from the main text and then it was rotated on the *xz*-plane (with a rotation point at its output surface) from 0° to 30° with a step of 3°. With this setup, the numerical results of the power enhancement on the *xz*-plane considering rotation angles in steps of 6° are shown in Figure S4a-f. For completeness, a schematic representation of the rotated plasmonic lens is shown at the bottom of each panel, respectively.

As observed, for angles smaller than 15° there is little impact on the power enhancement, as corroborated in Figure S4g where the power enhancement at the focal position is shown considering the full range of rotation angles. For larger angles (above 15°), the power enhancement significantly decreases to ~1.2. For completeness, we provide in Figure S4h the (*x*,*z*) coordinates of the focal position as a function of the rotation angle of the plasmonic meniscus lens. As observed, the position is shifted almost linearly with a small overall variation of ~$\Delta\lambda_0$= $0.35\lambda_0$ and ~$\Delta\lambda_0$= $0.4\lambda_0$ along the *x*- and *z*-axes, respectively, for rotation angles from 0° up to 30°. These results demonstrate that the proposed plasmonic meniscus lenses are robust in terms of the incident angle of SPPs for values smaller than 15°.

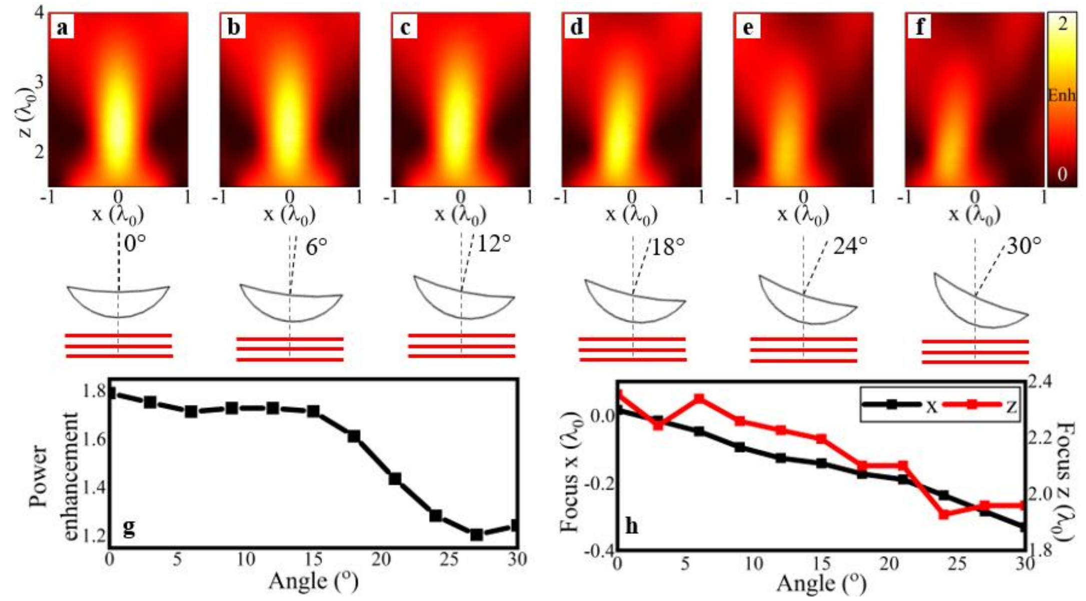

Figure S4. (a-f) numerical simulation results showing the power enhancement on *xz*-plane at y = 0 for a plasmonic meniscus lens as the one studied in Figure 5 from the main text when it is rotated on the *xz*-plane with angles from 0° to 15° with a step of 6°. A schematic representation of the rotated plasmonic meniscus lens is shown at the bottom of each panel, respectively. (g) Effect of changing the rotation angle on the power enhancement at the focus. (h) Effect of the rotation angle of the plasmonic meniscus lens on the focal position in *x* (black) and *z* (red) coordinates.
